# Socioeconomic outcomes of agricultural land use change in Southeast Asia

**DOI:** 10.1007/s13280-022-01712-4

**Published:** 2022-02-18

**Authors:** Jonas L. Appelt, Diana C. Garcia Rojas, Peter H. Verburg, Jasper van Vliet

**Affiliations:** 1 Institute for Environmental Studies, VU University Amsterdam, NU Building, De Boelelaan 1111, 1081 HV Amsterdam, The Netherlands; 2 School of Business and Economics, VU University Amsterdam, De Boelelaan 1105, 1081 HV Amsterdam, The Netherlands; 3 Swiss Federal Research Institute WSL, Zürcherstrasse 111, 8903 Birmensdorf, Switzerland

**Keywords:** Land use change, Large-scale land acquisitions, Smallholder agriculture, Sustainable development goals, Systematic review, Trade-off

## Abstract

Agricultural land use is transforming rapidly in Southeast Asia, often supported by development policies aiming primarily at economic growth. However, the socioeconomic outcomes of these changes for smallholder farmers remain unclear. Here, we systematically review cases of agricultural land use change in Southeast Asia to assess their socioeconomic outcomes and potential trade-off and synergies in these outcomes. Of the 126 reviewed cases, we find mostly positive outcomes for income (SDG 1, 100 cases) and employment (SDG 8, 11 cases), while outcomes on health (SDG 3, 9 cases) were mixed, and outcomes for food security (SDG 2, 44 cases), gender equality (SDG 5, 13 cases), and economic equality (SDG 10, 14 cases) were mostly negative. Studies describing multiple outcomes show indications of synergies between income and food security, and between income and employment, but also potential trade-offs between income and economic equality. In addition, we find that economic land concessions result in multiple negative outcomes more often than other types of land governance regimes. The results provide evidence that economic gains from agricultural land use change often come at a cost of other dimensions of sustainable development.

**Supplementary Information:**

The online version contains supplementary material available at 10.1007/s13280-022-01712-4.

## Introduction

Agricultural land use change is a dominant transformational trend in Southeast Asia, with different types of land use change taking place concurrently. These changes are manifested, for example, in the farming systems of smallholders, including changes from subsistence toward market-oriented agriculture (Rerkasem 2005; Zeng et al. 2018) and intensification of swidden systems (Dressler et al. 2017). In addition, several countries in the region have seen an increase in the area of economic land concessions (ELCs), which are typically large to very large in scale, and managed as businesses rather than traditional family farms (Hall 2011; Nolte et al. 2016). These ELCs sometime replace existing production landscapes, dominated by a mosaic of smallholder fields, orchards, and forests, but often also lead to the expansion of agricultural land into unused forest areas (Hurni and Fox 2018; Andrianto et al. 2019; Davis et al. 2020).

Changes in agricultural land use are often driven by economic incentives of farmers, in addition to motivations for ensuring their own survival (Malek et al. 2019). Empirical evidence also confirms that agricultural land uses change can increase household incomes, for example as a result of intensification of swidden areas (van Vliet et al. 2012). As agriculture is also important for the national economies of most countries in Southeast Asia, land use is instrumental for policies aiming at improving local and national development and reducing poverty. The eradication of swidden agriculture, for example, and the promotion of permanent agriculture has been supported by the government of Lao PDR by granting of land titles, in order to reduce rural poverty and induce economic development (Lestrelin et al. 2012). Similarly, Indonesia’s land policies have promoted economic development through oil palm plantations, both for smallholder farmers and by supporting large-scale land development of private companies (Rist et al. 2010; Obidzinski et al. 2012; Gatto et al. 2015). Governments granting ELCs to agribusinesses and investors often consider this an important investments in the agricultural sector, initiating businesses in the value-chain and creating employment (Arezki et al. 2015).

Land systems are essentially socio-ecological systems, which means that land use changes have both environmental and socioeconomic impacts. As a result, there is an increasing notion that land use is part of the solution for a broad range of the sustainability challenges we are facing today (Turner et al. 2021). This is especially true in the Global South, including Southeast Asia, where a large share of the population engages at least partly in agricultural production to support their livelihoods. Agricultural land use change has the potential to provide direct benefits for farmers, for example in terms of income and poverty, food security, and a number of other dimensions of human wellbeing (Ehrensperger et al. 2019; Abraham and Pingali 2020). However, while environmental impacts of land use changes, including impacts on biodiversity, hydrology, and biochemical processes, have been synthesized in a number of review studies, much less is known about their socioeconomic impacts (van Vliet et al. 2016).

With the establishments of the SDGs, the 2030 Agenda for Sustainable Development lay out a framework for assessing and comparing progress in multiple dimensions of human and societal development (Nilsson et al. 2016). The SDG framework provides an approach for combining a theoretical understanding of wellbeing (Coulthard et al. 2018) with global policy targets and local outcomes. As a socio-ecological system, land use is elementary for a range of different SDGs, making the SDG framework useful for analyzing different outcomes of land use change (Ehrensperger et al. 2019). It is the purpose of the 2030 Agenda to make progress on all targets together, rather than making progress on the targets individually. Therefore, analyzing the relations between different SDGs, including both synergies and trade-offs, is inherent to using the SDG framework (Nilsson et al. 2016; Ehrensperger et al. 2019; Kroll et al. 2019). Several studies have investigated the relations between SDGs as a result of agricultural land use change, often finding potential trade-offs (e.g., Scherer et al. 2018; Kroll et al. 2019; De Neve and Sachs 2020). These trade-offs indicate that land use change can help to solve several sustainability challenges, but that it does not necessarily contribute positively to the achievement of all SDGs at the same time (Verburg et al. 2015). Specifically, while agricultural expansion as well as agricultural intensification often leads to economic growth, it is also frequently associated with negative environmental consequences (Sodhi et al. 2010; Laurance et al. 2014; Rasmussen et al. 2018). Trade-offs between outcomes of agricultural land use change have been observed between improved food security (SDG 2) and a loss of biodiversity (SDG 15), while climate action (SDG 13) and biodiversity (SDG 15) are found to often be synergetic (Ehrensperger et al. 2019). However, while the relations between socioeconomic outcomes of agricultural land use change and the environment have been studied widely [e.g., Heck et al. (2018), Ramankutty et al. (2018)], we know relatively little about the relations between the different socioeconomic outcomes of these land use changes. In particular, little is known about the trade-offs and synergies that potentially exist and to what extent these hamper progress toward sustainable development.

In this paper we analyze socioeconomic outcomes of agricultural land use change, and identify potential trade-offs and synergies between these outcomes. We conduct a systematic review of case studies that report outcomes at the household level, using the SDGs as a framework for identifying socioeconomic outcomes as well as the potential trade-offs and synergies between them. Our study focuses on Southeast Asia because it is a highly dynamic region in terms of agricultural change, including the development of smallholders as well as the emergence of large-scale land acquisitions, potentially affecting multiple dimensions of sustainable development (Rigg et al. 2016; Schoenberger et al. 2017).

## Materials and methods

We base our analysis on a systematic review of case study evidence on agricultural land use change as reported in peer-reviewed scientific literature. Following the method described in the PRISMA statement [Preferred Reporting Items for Systematic Reviews and Meta-Analyzes (Moher et al. 2009)], the review of case studies included the following steps: (1) Identification of eligibility criteria, (2) Systematic collection of cases from literature, (3) Coding of information, (4) Analysis of coded cases. In addition, we assessed to what extent the cases are representative for the whole region of Southeast Asia in terms of socioeconomic and biophysical location characteristics.

### Eligibility criteria

We systematically searched for case studies reporting on outcomes of local level agricultural land use changes in Southeast Asia. ‘Local level’ is defined here as the case study area being smaller than national level and Southeast Asia is defined as the countries that are members of the Association of Southeast Asian Countries (ASEAN). To be eligible, cases needed to include information on (1) empirically observed agricultural land use changes, and (2) socioeconomic outcomes for the local population (i.e., households or farmers) as a result of these land use changes. Agricultural land use changes here refer to changes in the area of agricultural land as well as changes in the land use management of existing agricultural areas, such as changes in crop types or in management intensity. Land use changes can be included in the studies either as changes of land use over time, or as changes between a specific land use activity or between groups of actors involved in land use under otherwise similar conditions (i.e., space–time substitution). Socioeconomic outcomes refer to impacts of agricultural land use change on the economic, social, or human aspects of the SDGs, as relating to local farmers’ livelihoods and wellbeing. The selection was restricted to cases published in peer-reviewed journals, describing land use changes starting no earlier than 1950, with socioeconomic outcomes presented at household or farm level.

### Case study collection

Case studies were identified by systematically searching in Web of Science for papers published in the period January 2000 to December 2019, and including keywords identifying land use change processes (TOPIC = “land use” OR “land-use” OR ((agricultu* OR land) AND (*intensification OR extensification OR expansion OR abandonment OR diversification OR practice* OR transition OR transform*)) OR “land sharing” OR “land sparing” OR plantation OR concession OR “land grab*” OR resettlement). Moreover, we restricted the geographical area as described in the title or abstract to areas within ASEAN (TOPIC = (brunei* OR cambodi* OR indones* OR lao* OR malay* OR myanm* OR burm* OR philippin* OR filipin* OR singapore* OR thai* OR vietnam* OR “viet nam*” OR mekong OR sumatra OR java OR borneo OR kalimantan OR sulawesi OR luzon OR mindanao). The search string was checked against an initial set of 16 papers identified by the authors as a representative range of potential cases for inclusion, to ensure all cases appeared in the search result (Appendix S1). As this was the case, this was taken as an indication that our search string was sufficiently broad to return a range of relevant papers.

A subset of the eligible articles included information on multiple cases of land use change. In these instances, the cases were coded separately (i.e., multiple cases were coded from the same article). A case was defined as an area or group of actors for which the article analyzes land use changes and their socioeconomic outcomes in itself, without aggregating the results with other areas/groups.

To ensure consistency, a second reviewer independently checked a subset of the selection. This check initially resulted in a consistency of 70%, and discrepancies between both reviewers were discussed and resolved until complete agreement was achieved, and thus ensuring our criteria were unambiguous.

### Coding of cases

Cases were coded in terms of the publication characteristics, the case characteristics, the type of agricultural land use change, the land governance regime under which these changes take place, and their socioeconomic outcomes. The recorded information on the publication includes author, publication year, title, and the journal. The information on case characteristics includes the time period covered (or year of data collection), location, method for obtaining land use data, and the resulting crop system (rice, oil palm, rubber, aquaculture, conventional forestry/tree plantations, agroforestry, other specific crop types, or mixed/unclear crop composition).

Land governance regimes were coded as either (1) smallholder development, (2) development of economic land concessions (ELCs), (3) land conservation measures, or (4) resulting from state policies. ‘Smallholder development’ refers to cases where smallholder farmers decided on changing their land use or land management activities by themselves. While these decision are likely affected by a myriad of drivers, including also policies, the main agency for making land use decisions in this land governance regime is with the smallholder farmer (Debonne et al. 2021). The other three land governance regimes are characterized by agency lying primarily outside the farmers’ households. Specifically, ‘ELC development’ is affected mainly by actors in agricultural commodity chains, ‘land conservation’ refers to changes inspired by non-governmental organizations (NGOs) or governmental institutions, and ‘state policies’ come from state actors and include e.g., forest policies and policies restricting the use of shifting cultivation/burning of fallow areas (Debonne et al. 2021). Land governance regimes were interpreted as mutually exclusive classes, and coded based on the information and narrative provided by the authors of the papers in which the cases were found (further information on interpretation of categories in Appendix S1).

Agricultural land use changes where coded as land expansion, land contraction, intensification, and disintensification, following other comparable studies (van Vliet et al. 2015). Intensification is here understood as a change in inputs (e.g., increased fertilizer/pesticide inputs or increased mechanization), while disintensification denotes an opposite change. Transitions from shifting cultivation toward permanent agriculture were thus interpreted as an intensification process, unless specific other element were also described (e.g., abandonment of existing agricultural area).

Socioeconomic outcomes of land use changes were interpreted in terms of the SDGs and coded in general groupings that directly relate to these, including income and poverty alleviation (SDG 1), increasing food security (SDG 2), health outcomes (SDG 3), gender equality (SDG 5), employment (SDG 8), and economic equality (SDG 10). Only changes related to socioeconomic SDGs were coded, while outcomes related primarily to environmental SDGs were not included. Other analyzed socioeconomic SDGs were not excluded a priori but were not reported in any of the eligible case studies. Outcomes were interpreted as being either “positive” or “negative”, based on how they contribute to achievement of the respective goal (e.g., both a reduction in poverty and an improvement in income have been coded as positive for SDG 1). In addition to positive/negative, some outcomes were coded as “no-change” (only if actually described in the study) or as “unclear/multiple directions” if a case described both improvements and deteriorations related to a particular SDG, but with no overall trend. A more detailed quantification based on the sample size or quality of each case was not possible, due to the large variability in methods and the scientific fields of the included studies. The contribution of the socioeconomic outcomes of agricultural land use changes to the respective SDGs are in this paper based on a general understanding of the overall aim of each goal, i.e., outcomes of each case have not been linked to specific targets or indicators in the SDG framework.

### Analysis of coded cases

The analysis of socioeconomic outcomes of agricultural land use changes was based on counting outcomes of all cases per SDG, as well as for each type of agricultural land use change and each type of land governance regime separately. Relations between different socioeconomic outcomes were subsequently analyzed based on studies that record two or more types of outcomes. Subsequently, following the Agenda 2030 emphasis on making progress on all targets together, rather than on individual ones, we analyzed the relation between different socioeconomic outcomes of agricultural land use change. Where possible, the cases were analyzed to find positive synergies (case resulting in multiple positive outcomes), negative synergies (case resulting in multiple negative outcomes), and trade-offs (case resulting in both positive and negative outcomes). A few cases include mixed/no-change outcomes, which was interpreted positively or negatively according to the other outcome.

### Analysis of case study representativeness

The location of each case was recorded in the coding, in order to analyze the geographical distribution of the cases. Possible bias in the cases included in the review was investigated using representativeness analysis (Malek et al. 2019). The locations of cases were compared to the entire study area (Southeast Asia) in terms of the distribution of a number of social/human factors (distance to roads, gross domestic product, market access, population density, poverty, rural population), biophysical features (altitude, precipitation, slope, temperature), and of tree cover in the entire study region. Data representing these factors (Table 1) were resampled to a 10 × 10 km resolution. We used a Kolmogorov–Smirnov test, to assess the hypothesis that our cases are representative for the entire study area.

**Table 1:** Data used for the representativeness analysis

Variable	Description	Original resolution	Source
Altitude	Elevation above sea level, m	1 × 1 km	Hijmans et al. (2005)
Precipitation	Annual precipitation, mm	1 × 1 km	Hijmans et al. (2005)
Slope	Derived from altitude, degrees	1 × 1 km	Hijmans et al. (2005)
Temperature	Average temperature (mean of monthly means), degrees Celsius	1 × 1 km	Hijmans et al. (2005)
Distance to roads	Average distance to nearest road for points in cell, m	1 × 1 km	Derived from global road map (National Geospatial Intelligence Agency 2015)
Gross Domestic Product	GDP per capita in PPP, 2011 US$	Subnational units	Kummu et al. (2018, 2020)
Market accessibility	Indicator for the accessibility to markets, Index value	1 × 1 km	Verburg et al. (2011)
Population density	Distribution of human population, People/km^2^	1 × 1 km	CIESIN (2017)
Poverty	Share of population below national poverty line, %	1 × 1 km	Elvidge et al. (2009)
Tree cover	Canopy cover of vegetation taller than 5 m, percentage of cell	30 × 30 m	Hansen et al. (2013)

## Results

### Case study characteristics

The systematic search in Web of Science returned 8291 articles from peer-reviewed journals. Based on a screening of title and abstracts, we reduced this to 628 potentially relevant papers for further review, and based on the full texts we identified 114 papers eligible to be included in the review. The 114 papers cover 126 cases (the full list of papers, as well as their coding, is included in Appendix S1). In 77 cases changes are studied as change over time (average studied period is 13.9 years, with the shortest being 1 year and the longest being 48 years), while 49 cases use space–time substitution. The majority of the papers base their results on interviews with farmers or households (84%), while a minority of the papers use remote sensing (17%), field observations (14%), document analysis (6%), and other methods (7%).^1^ Several papers combine multiple methods, and therefore the sum of these percentages exceeds 100%.

**Fig. 1:**
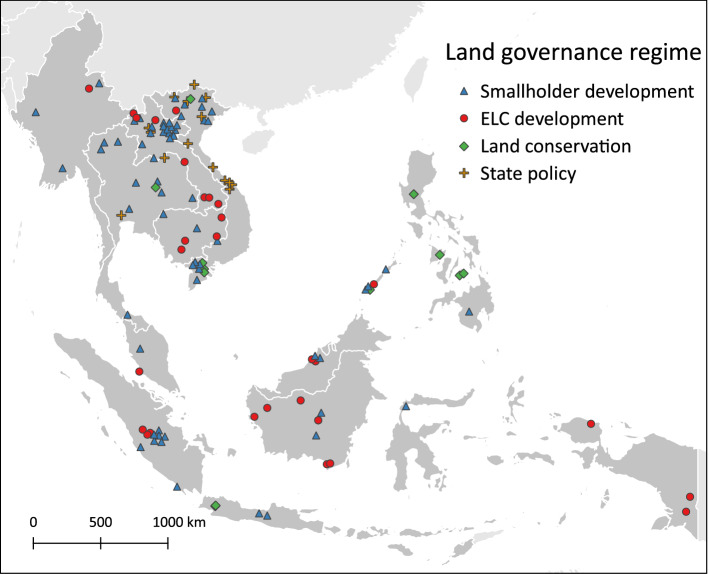
Location of cases and the land governance regime

The 126 cases included in the review are distributed over most of the countries in the study region, with the majority of cases found in Vietnam (30), Indonesia (30), and Lao PDR (28). Thailand is included with a medium number of cases (12), while fewer cases are found in the Philippines (9), Cambodia (7), Malaysia (6), Myanmar (4), and none are found in Brunei (Fig. 1).

### Observed agricultural land use changes and land governance regimes

The majority of the cases report changes related to smallholder development (67 cases, 53%, Fig. 1). Cases included as smallholder development typically describe a change toward a particular crop type, such as rice (17 cases, with 1 case describing a combined rice-aquaculture system), oil palm (8 cases), rubber (7 cases), and maize (6 cases). Another 31 cases (25%) describe the influence of ELCs on households living in or near one of these concessions. The majority of these cases describe changes toward perennial tree crops, including oil palm (14 cases), rubber (9 cases), and combined oil palm and rubber (3 cases). Of the 15 cases (12%) where land use changes were a result of state policies, most relate to either policies on reduction in shifting cultivation (5 cases) or to the development of urban areas (4 cases). For 13 cases (10%), land conservation was the main land governance regime affecting agricultural land use changes, including 4 cases describing conservation through various aquaculture activities.

Most of the included cases (88) can be described as an intensification of agricultural land use practices, while 35 cases describe expansion of agricultural land (Table 2). Intensification cases include changes such as an increase in the number of harvests, a shortening of the fallow period, and increase in fertilizer or pesticide application, while expansion refers to the development of agricultural activities on land that was previously non-agricultural. Decrease in human impact (disintensification and contraction) is considerably less prevalent as this is observed in only 20 cases (11 with disintensification, 9 with contraction). Cases with disintensification primarily relate to changes resulting from land conservation methods. These include transitions toward organic production [e.g., smallholder farmers going from production of conventional to organic tea in Northern Vietnam (Nguyen et al. 2018)], and multiple studies on the impact of decreasing pesticide use by diversifying mono-crop rice systems into combined rice-fish systems in the Mekong Delta in Vietnam (Berg et al. 2017; Tran et al. 2018). All cases reporting agricultural land contraction, report contraction as a result of state policies. Five of these cases relate to policies on reducing swidden, while in the other four cases contraction of agricultural area is a result of forced abandonment due to urban expansion [for example for Hue in central Vietnam (Nguyen et al. 2017)].

**Table 2:** Distribution of land use changes over the different governance regimes. Land governance regimes are considered as mutually exclusive, while multiple types of land use changes can be reported in one case

Agricultural land use changes	Land governance regime				
	Smallholder development	ELC development	Land conservation	State policies	Total
Intensification	52	20	5	11	88
Expansion	16	19	0	0	35
Disintensification	3	0	8	0	11
Contraction	0	0	0	9	9

Several cases describe multiple land use changes taking place in parallel. In particular, 12 cases observe expansion and intensification taking place in the same study area. This includes a number of cases with ELCs, where the establishment of large-scale plantations include both areas with prior smallholder production and new agricultural land gained through deforestation, as seen in the establishment oil palm plantations on Carey Island, Malaysia (Lai 2011), and of rubber plantations in southern Lao PDR (Kenney-Lazar 2012). In addition, five of the cases describing contraction of agricultural land also include a description of further intensification of the remaining agricultural area [e.g., government policies on restricting shifting cultivation in northern Lao PDR leading to shortening of fallow periods (Lestrelin and Giordano 2007)].

### Socioeconomic outcomes of agricultural changes

The most common socioeconomic outcome noted by the reviewed studies is a change in poverty or in the income level of households (SDG 1, 100 cases), followed by changes in food security (SDG2, 44 cases), economic equality within the community (SDG 10, 14 cases), gender equality (SDG 5, 13 cases), employment levels (SDG 8, 11 cases), and change to health-related factors (SDG 3, 9 cases). Table 3 shows that agricultural land use change impacts on poverty alleviation and income, employment, and health are predominantly positive, while impacts on food security, gender equality, and economic equality are mostly negative.

**Table 3:** Cases distributed on the socioeconomic outcomes recorded

Type of socioeconomic outcome	No. of cases	Negative (%)	No-change/mixed (%)	Positive (%)
SDG 1 - Poverty alleviation/income	100	13	15	72
SDG 2 - Food security	44	61	9	30
SDG 3 - Health	9	33	22	44
SDG 5 - Gender equality	13	62	15	23
SDG 8 - Employment	11	–	–	100
SDG 10 - Economic equality	14	71	21	7

**Fig. 2:**
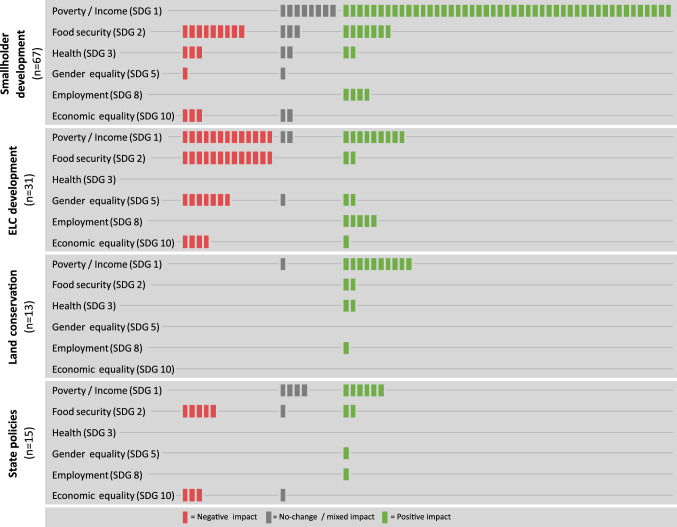
Distribution of socioeconomic outcomes over the different land governance regimes. Each square represents one case

When land use change outcomes are differentiated by their land governance regime, we find marked differences (Fig. 2). The impacts of smallholder development on the income and employment in rural households is predominantly positive. However, the impacts on food security and health are mixed. Economic equality, where reported, decreases because only some households can benefit from development opportunities, and these are typically not the poorest farmers, but the wealthier families instead [as is the case with expansion of cassava production in frontier villages in Cambodia, (Kurashima et al. 2014)].

ELC development mainly leads to negative outcomes, in terms of income and poverty alleviation, food security, and gender equality. These negative outcomes often relate to ELCs constraining the livelihoods of local households including changes toward non-agricultural livelihoods. For example, when land concessions are introduced, they limit the resources available for the local households, as is seen with the expansion of foreign owned rubber plantations in Luang Prabang Province in Lao PDR (Friis et al. 2016). ELC development also sometimes leads to positive outcomes in terms of income, employment, and food security, typically related to opportunities for smallholders provided by the ELC. For instance in cases where smallholders can participate through outgrower schemes (Feintrenie et al. 2010). Only one study reported a positive outcome on economic equality, showing a reduction in the Gini coefficient for income in the local community after a project promoting rubber and oil palm plantations in degraded areas in South Kalimantan (Hiratsuka et al. 2019).

Studies of changes under land conservation regimes report one case with neutral (mixed/no-change outcome) and otherwise only positive socioeconomic outcomes, predominantly in terms of income. Several of these studies report cases where specific agricultural practices are promoted by an outside entity e.g., an NGO. An example of this is the Landcare Programme in the Philippines that promoted various land conservation techniques, including use of natural vegetation strips to avoid soil erosion. After hillsides were stabilized, the project yielded a positive economic impact for the included upland households (Newby and Cramb 2011).

State interventions yield mixed socioeconomic impacts, mainly depending on the type of land use change that takes place under this land governance regime. Urban expansion has in some cases led to an increase in income and employment (Thi et al. 2019). However, the loss of agricultural land can also lead to loss of food self-sufficiency, impacting food security negatively for some households (Nguyen et al. 2017).

**Fig. 3:**
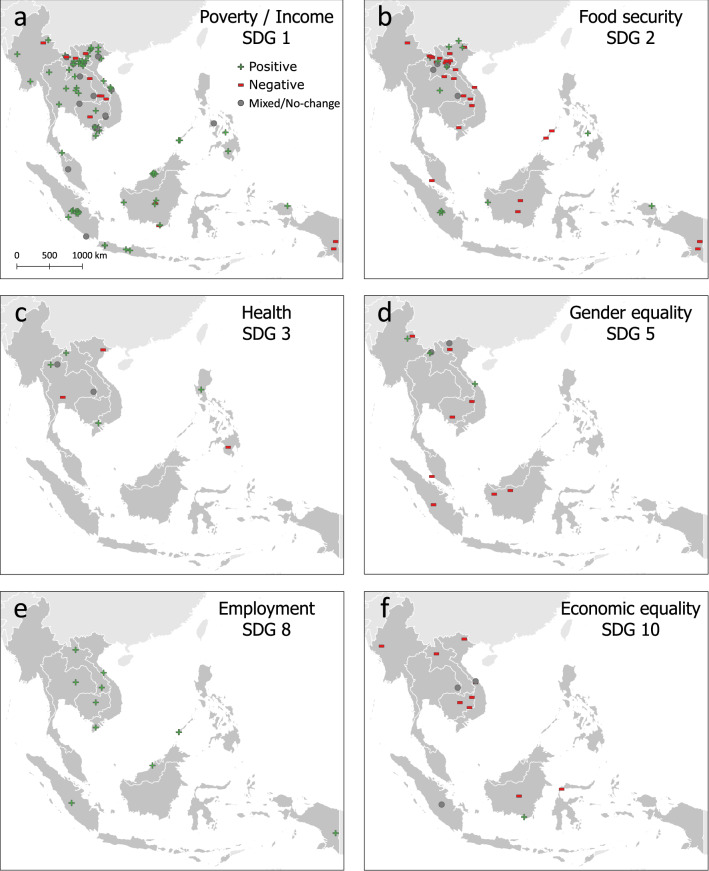
Location of cases according to type of socioeconomic outcomes. Green plus represents a positive outcome, red dash represents a negative outcome, and gray circle represents a neutral outcome (either a “no-change” outcome or mixed/unclear outcomes)

Positive and negative impacts are not spread evenly over the study area for the different socioeconomic impacts. Cases reporting positive outcomes on income and poverty alleviation are spread over the entire study region, while the majority of negative outcomes are concentrated in Lao PDR (Fig. 3). Food security shows a similar clustering of negative cases in the Lao PDR, but with additional negative cases in other areas, including Kalimantan, Indonesia, and on Palawan in the Philippines. Cases reporting on health, gender equality, employment, and economic equality are spread more evenly, and the small number of cases do not reveal clear clustering patterns.

### Relations between socioeconomic outcomes

**Fig. 4:**
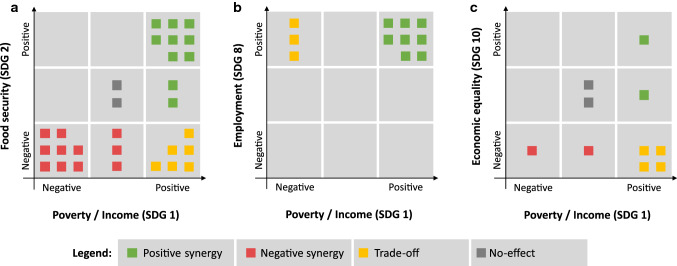
Trade-offs and synergies between reported socioeconomic outcomes. Squares represent individual cases that report multiple SDG outcomes, thus allowing to assess their relation explicitly. We only show results for SDG pairs that are jointly reported in more than ten cases

The combination of all cases indicates that smallholder development leads to mainly positive outcomes in terms of income (SDG 1) and employment (SDG 8), and mixed to negative outcomes on food security (SDG 2), health (SDG 3), gender equality (SDG 5) and economic equality (SDG 10) (Fig. 2). This suggests a trade-off exists between the primarily economic aspects (SDG 1 and SDG 8) and the other socioeconomic outcomes of these agricultural land use changes. However, studies that analyze multiple outcomes per case report positive synergies and negative synergies more often than trade-offs (Fig. 4).

Cases that report on both income (SDG 1) and food security (SDG 2) in the same study show that agricultural land use change result in largely synergetic outcomes (Fig. 4a). A total of 29 cases report on both of these outcomes, with 10 of these showing a positive synergy, and 11 showing a negative synergy. Six cases show a trade-off between income and food security, all of which report an increase in income and a decrease in food security.

Changes in both income (SDG 1) and employment (SDG 8) were jointly reported in eleven cases (Fig. 4b). Eight cases show a positive synergy between these two outcomes, while three cases report a trade-off with an increase in employment and negative development in relation to poverty/income. The three cases showing trade-offs all relate to ELC developments, including large-scale concessions for oil palms in Indonesia (Obidzinski et al. 2012), and in Lao PDR for rubber (Friis et al. 2016) and general land development (Delang et al. 2013). In these cases, the land concessions have increased the availability of off-farm employment, while at the same time having a negative impact on smallholder agriculture, leading to a net loss in income for the local households.

Cases reporting outcomes on both poverty/income (SDG 1) and economic equality (SDG 10) show both trade-off and synergies (Fig. 4c). Four of the cases describe a trade-off, with improvement in income and a decrease in the local economic equality, while another four cases are equally distributed over positive and negative synergies. Other combinations of socioeconomic impacts were only studied in fewer than ten cases, which was deemed insufficient to detect a trend in their relation.

### Representativeness of cases

In order to assess the representativeness of the cases included in our review we compared a number of biophysical and social/human characteristics of our sample with those of the entire study region. There is no significant difference between the spatial distribution of biophysical characteristics across the case studies and the distribution of these characteristics across the entire study region, except in terms of precipitation: Cases in the sample have on average a lower precipitation (Table 4). However, in terms of social/human characteristics there is a significant difference between the case areas and the region as a whole for all characteristics except for poverty (Table 4). Specifically, the included case studies are on average closer to the road, with a lower GDP per person, a lower market accessibility, and a higher population density, than the entire region. Also, the case study locations have on average a lower tree cover than the total study area. These deviations show that cases are more often located in agricultural areas that are some distance from traditional urban centers (market distance), but with decent infrastructure connections (distance to road), and internal pressures on agricultural development (low income and high population).

**Table 4:** Results of Kolmogorov–Smirnov test. *p* values indicate the significance of the difference between the reviewed case studies and the entire study area; values < 0.05 (*) indicate that the case study locations are significantly different from the entire region for the specific variable

Type	Variable	*D* value	*p* value
Biophysical characteristics	Altitude	0.083	0.353
	Precipitation	0.225	0.000*
	Slope	0.068	0.613
	Temperature	0.113	0.083
Social/human characteristics	Distance to roads	0.291	0.000*
	Gross Domestic Product	0.220	0.000*
	Market accessibility	0.151	0.006*
	Population density	0.176	0.001*
	Prevalence of poverty	0.093	0.229
Land use/cover characteristics	Tree cover	0.142	0.013*

## Discussion

### Socioeconomic outcomes of agricultural land use change and their relations

We find that most of the documented cases of agricultural land use changes in Southeast Asia have led to an improvement in local income (SDG 1) and in employment (SDG 8), but have also led to predominantly negative outcomes on food security (SDG 2), gender equality (SDG 5), and economic equality (SDG 10), and to mixed results on health outcomes (SDG 3). These results nuances the understanding that agricultural land use change predominantly leads to positive socioeconomic outcomes as reported elsewhere [e.g., Meyfroidt (2018)]. The positive socioeconomic impacts relate primarily to changes in income and economic development, while other types of socioeconomic impacts are frequently not assessed at all (Joffre et al. 2017). Positive impacts on income for smallholders are not surprising, as agricultural land use changes are typically motivated by a desire to increase income (Malek et al. 2019). At the same time, an increase in income can hide a more complex socioeconomic situation, as in many cases wealthier farmers are better able to capture gains from land use changes, thereby increasing local inequality. In such cases the land use change can yield positive outcomes in income on a household level, for few selected farmers, but negative outcomes on a community level due to increased inequality (Kurashima et al. 2014; Vicol et al. 2018).

Socioeconomic outcomes of agricultural land use change differ markedly across the different land governance regimes. Smallholder development, which is studied most frequently in the reviewed cases, largely follows a pattern with mostly positive outcomes for poverty alleviation and employment, mixed outcomes for health and food security, and mostly negative outcomes for gender equality and economic equality. Cases with ELCs, on the contrary, report more negative than positive outcomes for poverty and income, as well as for food security and gender equality, while health outcomes are not studied at all. Negative impacts of ELCs on local income generation seems to stem from displacement and reduction in the access to land and water resources in the impacted areas, including forced displacement of the original farming households [e.g., Kenney-Lazar (2012)]. At the same time, some cases report positive impacts of ELC development on incomes, due either to creation of employment opportunities on the ELC (Shively and Pagiola 2004; Obidzinski et al. 2012) or from joint ventures and outgrower schemes for smallholders located close to the ELC (Feintrenie et al. 2010; Rist et al. 2010). The ambiguous, context-specific impacts of ELCs on the economic opportunities of local households’ has also been reported in previous reviews on land concessions, showing that the impacts of ELCs can depend on the existing land use regime (Oberlack et al. 2016), and can results in both winners and losers in the same local context (Oya 2013). Increased employment as a result of ELCs development relates to a continued process of rural diversification happening across Southeast Asia, where farming households expand their livelihoods by engaging in off-farm work, both inside and outside the agricultural sector (Rigg and Salamanca 2017). ELCs may provide a local source of income for households that otherwise might have sought additional income sources through rural–urban migration, though evidence for how ELCs impact migration patterns in the region is still developing (Kelley et al. 2020).

Changes under a land conservation regime stand out as most of these cases represent some form of disintensification, and these cases only have neutral and positive socioeconomic outcomes. Yet, while these positive outcomes are reported in relation to agricultural land use change, they are in some cases not causally related. Instead, both can be the result of additional training of farmers and increased knowledge dissemination (Tipraqsa et al. 2007; Newby and Cramb 2011). Land use changes as a result of state interventions differ more widely and include agricultural land contraction due to urban expansion (Nguyen et al. 2017; Thi et al. 2019), reductions in shifting cultivation (Lestrelin and Giordano 2007), and increases in the area under forest cover (Sandewall et al. 2010; Nguyen et al. 2016). As a result of the variation in observed land use changes, socioeconomic outcomes also differ, while the total number of cases do not provide a clear trend.

Cases that report on multiple socioeconomic outcomes report positive and negative synergies more often than trade-offs. The synergistic relation is strongest for the combination of food security and poverty alleviation/income, which indicates that areas with an increase in income generally also see a decrease in food insecurity, and vice versa (e.g., Souphonphacdy et al. 2012). However, six cases report a trade-off between income and food security. These cases all represent examples of transitions from subsistence toward market-oriented agriculture [e.g., Harrington (2015), Dawson et al. (2019), and Kallio et al. (2019)]. In these cases, the decrease in food security is a direct consequence of the agricultural land use change, as land is no longer used to produce food for the household, but instead it is used to produce cash crops such as maize and rubber to be sold at the market. ELC development stands out as it leads to negative synergies in multiple cases, mostly between food security and income. In other words, these agricultural land use changes affect farmers and their households negatively twice, as they lose part of their income as well as the capacity to produce their own food. This is notable, since ELCs are also often associated with significant environmental destruction (Andrianto et al. 2019; Davis et al. 2020). Compared to previous reviews of SDG interactions (Pradhan et al. 2017; Kroll et al. 2019), this highlights the importance of recognizing that synergistic relationship between SDGs also makes it possible for land processes to lead to negative synergies [see also Rasmussen et al. (2018)]. The number of studies reporting multiple socioeconomic outcomes was relatively small to the point that analyses of most pairs of SDGs was not possible. This confirms findings by Ehrensperger et al. (2019) that show that the land system science community disproportionally focuses on a small number of trade-offs between SDGs, often including either SDG 13 (Climate action) or SDG 15 (Life on land).

Our results are based on a sample of case studies from the literature that is not necessarily representing the full scope of agricultural land use in Southeast Asia. The representativeness analysis suggests that our sample is biased toward areas with on average a lower GDP, a lower market accessibility, and a higher population density. These characteristics are typical for agricultural frontiers (Hirsch 2009), and while this might not be representative for all agricultural land, these are the locations where most land use change takes place. However, while frontier areas may show more prominent systemic changes to the agricultural system, it could mean that in the literature, the outcomes of more gradual land use changes in established agricultural regions are underrepresented. Moreover, a bias toward agricultural frontiers does not preclude that trade-offs also take place in other agrarian areas that are subject to slower changes. The nature of these impacts may be different in such areas, as the contextual conditions differ. Next to the geographic bias, this study also shows a clear bias in scope, preferring economic outcomes over other outcomes. This bias indicates a gap in the scientific literature, and it also suggests the somewhat uneven attention devoted to different SDGs, despite their design as a holistic set of goals (Nilsson et al. 2016; Weitz et al. 2018).

### Implications for sustainable development

The multiple socioeconomic outcomes of agricultural land use change as well as the relations between these outcomes further confirm the role of land use as a nexus for sustainable development (Verburg et al. 2015; Ehrensperger et al. 2019; Turner et al. 2021). Other studies have shown that a potential trade-offs exist between socioeconomic SDGs and environmental SDGs (Scherer et al. 2018), and that this trade-off can also exist for agricultural land use change in the Global South (Meyfroidt 2018; Rasmussen et al. 2018). This study complements these findings by nuancing the notion that development in agricultural land use primarily leads to positive socioeconomic outcomes. We find that agricultural land use change often leads to economic growth for the local population in terms of income generation (SDG 1) and employment (SDG 8), but that other aspects of human wellbeing may suffer, including food security (SDG 2), health (SDG 3), gender equality (SDG 5), and economic equality (SDG 10). It is noticeable that the SDGs that are studied less frequently, notably Good health (SDG 3), Gender equality (SDG 5), and Economic equality (SDG 10) often have negative outcomes. This suggests a blind spot in our knowledge of the outcomes of agricultural land use change.

Only few of the studies analyzed multiple socioeconomic impacts of the same land use change, and therefore only a few explicitly find trade-offs between the investigated SDGs (Vongvisouk et al. 2014; Harrington 2015; Dawson et al. 2019). In fact, cases that assess multiple socioeconomic impacts more often find positive and negative synergies than trade-offs. The negative synergies are especially worrisome, as these are found in relation to agricultural expansion and intensification (Kenney-Lazar 2012; Andrianto et al. 2019) thus also leading to potential negative environmental impacts.

Several land use policies in Southeast Asia aim for economic development, by incentivizing a shift toward more market-oriented agriculture, with products exported to urban centers either nationally or internationally. However, these policies are typically sector specific and do not include a more comprehensive view on sustainable development. This review shows that many scientific studies also limit their assessment of land use change impacts to one single or a few outcomes. Yet, some valuable exceptions exist (see e.g., Sampantamit et al. (2020) for an assessment of SDGs in relation to fisheries in Thailand and Dolley et al. (2020) for an assessment of trade-offs and synergies related to urbanization in China). At the same time, the combined evidence of the reviewed cases indicates that a narrow policy focus on developing market-oriented agriculture might result in detrimental outcomes in other aspects of sustainable development, including related to food security, health, gender equality, and economic inequality. Since we identified trade-offs between economic development and other socioeconomic outcomes both in specific studies and across a range of cases, we argue that there is a need for policies to integrate multiple different dimensions of sustainable development instead of focusing primarily on economic growth (Eisenmenger et al. 2020).

Findings of socioeconomic outcomes of agricultural land use change in Southeast Asia could also be relevant for other world regions, especially in areas with similar land governance regimes. A global analysis of land use decision making indicates that decision makers in Southeast Asia are predominantly motivated by survival and livelihood objectives (Malek and Verburg 2020). This analysis further indicates that land use decision makers are most likely characterized as survivalists and market-oriented smallholders in our study region (Malek et al. 2019; Malek and Verburg 2020). Both these objectives and types of decision makers are also prevalent in South Asia and Sub-Saharan Africa, suggesting that socioeconomic outcomes as well as the trade-offs and synergies found in this study might also occur in these regions. Nonetheless, land use change processes and their outcomes are at least partly context dependent, limiting the transferability of findings from this study to other world regions. For example, the average yield gap for cereals is higher in Sub-Saharan Africa than in Southeast Asia (Mueller et al. 2012), which could indicate that smallholders in Sub-Saharan Africa might benefit more from intensification than we have found in this study (van Ittersum et al. 2016; Holden 2018). Martin et al. (2018), on the other hand, notes that poorer farmers have a limited ability to capture the gains from agricultural intensification, which suggests that intensification might not be an ideal option for smallholders in poorer countries, including some areas of Sub-Saharan Africa.

ELCs are also frequently observed in Sub-Saharan Africa, as well as in large parts of Latin America (Debonne et al. 2021; The Land Matrix 2021). While Latin America is rather different from Southeast Asia in terms of the types of smallholder systems observed (Malek and Verburg 2020), this further confirms the potential relevance of our findings for Sub-Saharan Africa. Consistent with our findings, global assessments on ELC impacts have also highlighted the potential negative consequences for the local populations in relation to loss of income (Davis et al. 2014) and food security (Müller et al. 2021). A review ELC case studies in Africa also shows how local consequences in this region are mostly negative (Hufe and Heuermann 2017). Hence, even though the local context can vary between local cases, our findings as well as these studies all show that ELC developments often result in negative synergistic socioeconomic outcomes.

## Conclusions

In this study, we find that agricultural land use changes in Southeast Asia generally result in improvements in income and employment, but a worsening of other socioeconomic areas, notably health, food security, gender equality, and economic inequality. Case study evidence, thus, suggests that ongoing changes in the region, primarily processes of intensification and expansion on agricultural land, might not lead to improvements across all SDGs. Development of ELCs can be especially problematic, with multiple examples of ELCs resulting in negative impacts on a range of socioeconomic SDGs (negative synergies). These findings show the need for development policies and scientific studies to go beyond economic wellbeing and include a broader range of socioeconomic outcomes as a result of agricultural land use change.

## Supplementary Information

Below is the link to the electronic supplementary material.

Supplementary file1 (XLSX 104 kb)

## Footnotes

1 Other methods cover a variety of different methodologies, including key-person interviews, literature studies, participant observations, self-reporting from research subjects and others.
